# Important Approaches to Enhance Reverse Osmosis (RO) Thin Film Composite (TFC) Membranes Performance

**DOI:** 10.3390/membranes8030068

**Published:** 2018-08-21

**Authors:** Ahmed Al Mayyahi

**Affiliations:** Department of Chemical Engineering, University of Missouri, Columbia, MO 65211, USA; aaakz5@mail.missouri.edu or sumrabm@yahoo.com

**Keywords:** nanoparticles (NPs), thin film composite (TFC), interfacial polymerization (IP), surface modification

## Abstract

Thin film composite (TFC) membrane, which consists of polyamide (PA) active film rests on porous support layer, has been the major type of reverse osmosis (RO) membrane since its development by Cadotte in the 1970s, and has been remarkably used to produce clean water for human consumption and domestic utilization. In the past 30 years, different approaches have been exploited to produce the TFC membrane with high water flux, excellent salt rejection, and better chlorine/fouling resistance. In this brief review, we classify the techniques that have been utilized to improve the RO-TFC membrane properties into four categories: (1) Using alternative monomers to prepare the active layer; (2) modification of membrane surface; (3) optimization of polymerization reactions; and (4) incorporation of nanoparticles (NPs) into the membrane PA layer. This review can provide insights to guide future research and further propel the RO TFN membrane.

## 1. Introduction

Because of the rapid growth of the world population and rising water needs, water shortage problems have become dominant [1,2]. In the last century, as the global population quadrupled, the world water demand has increased sevenfold. The problem of water scarcity is not only a problem of appropriate techniques, but also a social and educational problem relying on national and global endeavors as well as on technical solutions [3]. To address water shortage problems, many techniques have been developed including distillation, membrane reverse osmosis (RO), mechanical vapor pressure compression, electrodialysis and nanofiltration processes [4]. Membrane separation processes are gaining global acceptance in both water treatment and desalination due to their simplicity and relatively low cost compared to other treatment technologies [5]. RO membranes can be effectively used to remove salts and other pollutants from brackish water [6]. The water is transferred through the RO membrane by diffusion [7], while the salt is rejected by size exclusion and repulsion electrostatic force between the membrane surface and dissolved ions, which is caused by charge difference [8,9]. For efficient desalination, the membranes must be permeable to water, impermeable to solutes, and capable of tolerating high operating pressures [9].

The first work on RO membranes was initiated by Reid and co-workers [10] in the early 1950s, when they successfully fabricated an active cellulose acetate membrane to remove salt from water. The membrane exhibited efficient desalination (salt rejection: 96%), but water flux through the membrane was significantly low. Researches continued at the University of California, Los Angeles, with the concern of improving water flux without sacrificing salt rejection [11]. In 1961, Sourirajain [12] enhanced membrane flux by increasing cellulous film porosity through using pore-forming monomers. However, cellulose acetate membrane has limited applications due to its weak chemical resistance and low thermal stability [13,14]. Thus, many studies were conducted to produce a membrane with better characteristics. In 1979, Burns and coworkers [15] suggested the use of aromatic polyamide (PA) membrane, which is recognized by its cheap prize and high temperature tolerance, as an alternative [15,16]. Though water permeability by this membrane is less than that of cellulose acetate membrane, their salt rejection is higher.

**Figure 1:**
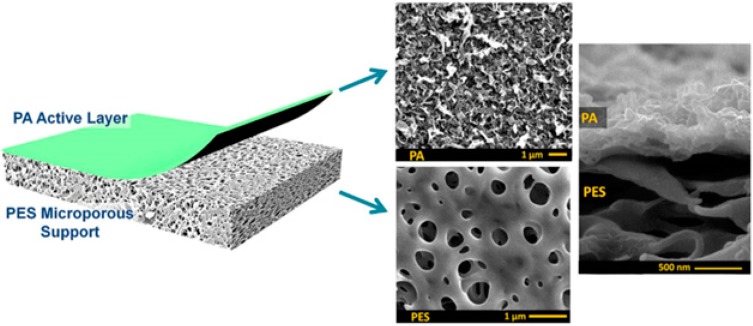
Polyamide thin film composite membrane, reproduced with permission from Khorshidi et al. [18].

A major breakthrough in the field of RO is the development of PA thin film composite (TFC) membrane. This membrane consists of two layers, the top is an active PA film prepared by the reaction between m-Phenylenediamine (MPD) and trimesoyl chloride (TMC) on a microporous polysulfone support (PSU), the bottom layer [17,18], as shown in Figure 1. Water flux through the composite membranes depends on the hydrophilicity of the membrane surface and the characteristics of the porous support layer, while salt rejection relies on the surface charges and PA structure [14].

Several state-of-the-art reviews have been published to highlight the types of RO-TFC membranes and their performance. For instance, Yin et al. [19] detailed the benefits of incorporating various nanoparticles on the membrane’s water flux, salt rejection, chlorine resistance, and antifouling properties. Another review by Xu et al. [20] discussed the influences of sub-layer adjustment on pressure gradient across the membrane and subsequent performance. Recently, Gohil et al. [21] reviewed the systematic development of TFC membranes with their structural composition and separation characteristics, including the effects of various additives and IP reaction parameters. However, until now, there has been no clear classification of the approaches that have been used to enhance RO-TFC membranes properties. Thus, the objective of this brief review is to fill this gap in literature and provide new insights for readers to improve their knowledge in this field.

## 2. Using Alternative Monomers to Prepare the Active Layer

Because membrane performance is substantially dependent on a thin film structure and its chemical properties, different monomers have been used to prepare the PA, as shown in Table 1. For example, Li et al. [22] used three different isomeric biphenyl acid chlorides (mm-BTCE, om-BTCE, op-PTCE) to react, separately, with m-phenylenediamine (MPD) on a porous support. Results indicated that the membrane prepared from op-PTCE exhibited higher water flux and lower salt rejection, while those prepared from mm-BTCE and om-BTCE showed lower water flux and higher salt rejection. The reason behind permeability enhancement could be due to the high density of the carboxylic acid group on the membrane prepared from op-PTCE, which led to better contact with water molecules. On the other hand, the higher salt rejection might be because of the thicker PA layer produced by using mm-BTCE and om-BTCE. Another study was reported by Wang et al. [23] in which introducing 3,5 diamino-*N*-(4-2-aminophenyl)-benzamide (DABA) as a monomer to react with TMC through interfacial polymerization resulted in a more hydrophilic, thinner, and smoother membrane.

One challenge facing RO TFC membrane durability is the degradation of the PA layer by chlorination [37]. Liu et al. [33] used three different polyacyl chlorides including 5-isocyanato-isophtahloyl chloride (ICIC), 5-chloroformyloxy-isophthaloyl chloride (CFIC), and TMC to prepare the TFC membrane with high chlorine tolerance. Results showed that the membrane prepared from MPD-CFIC and MPD-TMC possessed better chlorine stability when compared to MPD-ICIC membrane. It has been pointed out that the urea bond (NHCONH-) in MPD-ICIC could be easily attacked by chlorine. Recently, a composite membrane with high chlorine resistance has prepared through interfacial polymerization of hexafluoroalcohol (HFA)-aromatic diamine and trimesoyl chloride (TMC) [34]. The steric and electron withdrawing properties of HFA groups mitigated the probability of chlorine attack on the benzene rings or amide groups in the PA layer.

In term of fouling resistance, Hilal et al. [35,36] prepared composite membranes with improved antifouling properties by interfacial polymerization of bisphenol A (BPA) and trimesoyl chloride (TMC). This was attributed to the strong repulsion force between the negatively-charged bisphenol and organic foulants.

## 3. Modification of Membrane Surface

It was found that membrane performance is greatly affected by the treating steps that follow the synthesis process [38,39,40,41,42]. Chemical surface modifications are one of the promising post-treatment techniques that have been widely used to enhance TFC membrane surface properties. For example, Mickols and coworkers [43] used ethylenediamine and ethanolamine to increase membrane hydrophilicity. Their study showed that increasing the hydrogen bonding at the PA layer could enhance the interaction between water molecules and membrane surface, resulting in high water flux. Another study by Kuehne et al. [44] demonstrated that soaking the membrane in a solution containing glycerol promoted surface wettability and a 70% increase in water flux was obtained.

Wilf et al. [45] coated poly(vinyl alcohol) on TFC membrane surface to enhance fouling resistance and membrane durability. The modified membrane demonstrated better resistance against organic fouling when compared with the normal TFC membrane. Moreover, the membrane showed good permeability and long-term stability. The enhanced fouling resistance was ascribed to the lower rate of organics adsorption on the coated membrane. Coatings of poly(*N*,*N*-dimethylaminoethyl methacrylate) (PDMAEMA) have also exhibited enhanced chlorine resistance according to Kang et al. [46]. Additionally, Sakar et al. [47] used dendrimer-based coatings to reduce fouling effects. Recently, Ngo et al. [48] used redox initiated graft polymerization to coat TFC membrane with hydrophilic poly(acrylic acid). The coated membrane had lower roughness than the virgin one, and subsequently better fouling resistance and water flux was achieved.

**Figure 2:**
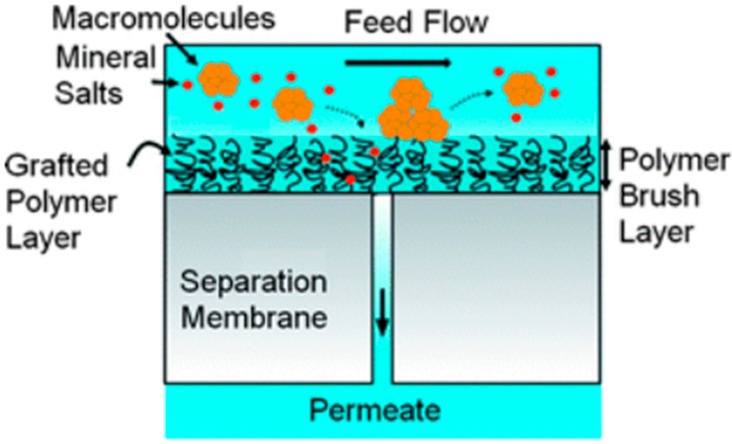
A schematic illustration of the nano-structured RO membrane, showing the antifouling polymeric brush, reproduced with permission from Lin et al. [51], with copyright permission from ©2010 Royal Society of Chemistry.

Wu et al. [49,50] used gas plasma treatment to modify the TFC membrane. More carboxylic groups were introduced onto the surface by oxygen gaseous plasma treatment, which resulted in high water flux. On the other hand, argon plasma treatment improved chlorine resistance by introducing more amide groups onto the membrane surface. In addition, Lin et al. [51] demonstrated that the antifouling properties of the TFC membrane could be improved by using atmospheric gas plasma treatment. This kind of treatment created a polymeric brush at membrane surface which was capable of mitigating the attachment of organic foulant, Figure 2. However, plasma-induced grafting is a promising approach to produce a membrane with significant performance; however, it has not been thoroughly investigated and further research in this area is required.

Bing et al. [52] used redox initiation to enhance TFC membrane performance, especially chlorine resistance. Immersing the membrane in potassium persulfate (K_2_S_2_O_8_) solution initiated the interfacial crosslinking between the active film and PSU support, producing a thinner PA layer with more functional groups and denser cross linking. Reducing PA thickness led to enhanced water flux, as the water spent a shorter time to penetrate the membrane. On the other hand, increasing the crosslinking improved salt rejection by narrowing the passages for salt transportation. Moreover, the crosslinking reduced the N-chlorination sites on the membrane surface and hence, improved chlorine resistance.

## 4. Optimization of Polymerization Reactions

A major area of intense research is the optimization of interfacial polymerization reaction mechanisms such as kinetics, solvent solubility, reactant diffusion coefficient, reaction time, polymer molecular weight range, and characteristics of micro-porous support [53,54,55,56]. Tomaschke et al. [57] found that mixing amine salts with the casting solution formed a cross-linked membrane with an improved rejection. Chau et al. [58] added *N*,*N*-dimethyleformamide into a casting solution that introduced more carboxylic functional groups to PA layer, and eventually increased water flux. Kwak et al. [59] used dimethyl sulfoxide as an additive to modify the aromatic PA thin-film layer. The quantitative analysis of the surface morphology showed correlation between water permeability and both surface area and surface roughness; the flux improved with increasing roughness and surface area without a significant loss of salt rejection. Other researches showed that the addition of ethers, sulphur compounds, and alcohol- or water-soluble polymers to casting solution could produce high permeability without jeopardizing salt rejection [60,61,62].

Instead of modifying the casting solution, Michol et al. [63,64] succeeded in adding a complexing agent (phosphate containing compound) to the poly functional acyl halide prior to the substantial reaction between functional acyl halide and poly functional amide. It was thought that the addition of a complexing agent resulted in the formation of “association” with a polyfunctional acyl halide monomer capable of reducing the hydrolysis of acyl halide functional groups and permitting sufficient subsequent reaction with amine functional groups, thus resulting in a significant enhancement in membrane performance.

Another alternative approach for optimizing the polymerization reaction is to introduce surface-modified macromolecules (active additives) to acyl halide solution. This approach depends on the concept that the macromolecules may transfer to the PA film surface during the polymerization and change surface properties of membrane whilst maintaining bulk properties unaltered [65,66]. Arafat et al. found that by using poly(ethylene glycol) as an active additive in the interfacial polymerization, the water flux and salt rejection were significantly increased [67].

## 5. Incorporation of Nanoparticles (NPs) into Membrane PA Layer

**Figure 3:**
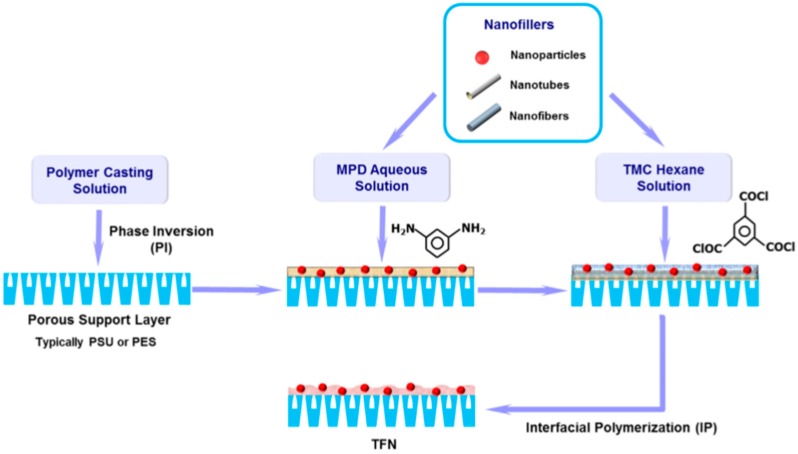
TFN membrane fabrication by the IP process, reproduced with permission from Yin et al. [19], with copyright permission from © 2014 Elsevier.

A new class of membrane has been formed by the incorporation of nanoparticles (NPs) into the top layer of conventional thin film composite membrane (fabrication process Figure 3). Table 2 summarized the performance of RO thin film nanocomposite (TFN) membranes that were reported in literatures and the next section discusses the most important studies.

Jeong and Huang [60] reported that adding NaA zeolite NPs into the PA could result in an increase in water permeability without decreasing salt rejection. It was claimed that the superior hydrophilicity, high negative surface charge, and internal pores of zeolite nanomaterial facilitated water absorption and movement across the membrane, while maintaining high salt rejection via Donnan exclusion.

Lind et al. [62] studied the influence of zeolite crystal size on the apparent structure, morphology, interface, and permeability of zeolite-PA TFN membranes. The existence of zeolite NPs resulted in higher permeability, greater negative surface charge, and thicker PA when compared with the raw membrane. The smaller NPs produced greater permeability, while the larger NPs produced more favorable surface properties. This study implied that the size of NPs may be considered an additional “degree of freedom” in designing the nanostructured membranes. Recently, Mayyahi [106] used quantum dots as an ultra-small filler to modify the TFN. Both water flux and salt rejection were increased upon the addition of QDs.

Another study by Fathizadeh et al. [74] showed that increasing MPD and TMC concentrations to 3% *w*/*v* and 0.15% *w*/*v*, respectively, during TFN preparation in the presence of zeolite NPs formed a membrane with superior water flux but declined NaCl rejection. The low solute rejection was attributed to the poor dispersion of nanoparticles in the high molecular weight PA layer. The aggregation of NPs could have generated micro-holes in the PA, which allowed the brackish water to pass through. The reported results suggested that the relation between NPs and IP condition is another important factor that needs to be addressed.

In addition to zeolite, different NPs such as nano-silica [68,71,104], multiwall carbon nanotubes [69,72], zwitterion functionalized-carbon nanotubes [78], Titanium dioxide (TiO_2_) [101,107], and clay nano-sheets [90] have been used to modify the composite membranes. All these researches showed that imparting NPs to the PA could enhance membrane performance in terms of permeate flux, salt rejection, chlorine resistance, and antifouling properties. For instant, Barona et al. [86] found that incorporating aluminosilicate single-wall carbon nanotubes (SWNTs) into the membrane surface resulted in a significant increase in water flux without affecting salt rejection. The functional groups on SWCNTs secured excellent dispersion of fillers in the PA and, as a result, enhanced the overall performance. A remarkable enhancement in membrane performance was achieved in another study by Jun et al. [76] upon the addition of MCM-41 silica NPs without compromising salt rejection. The high water permeability was ascribed to the enhanced membrane hydrophilicity as well as the pores in the NPs that imparted extra channels for water transportation.

**Figure 4:**
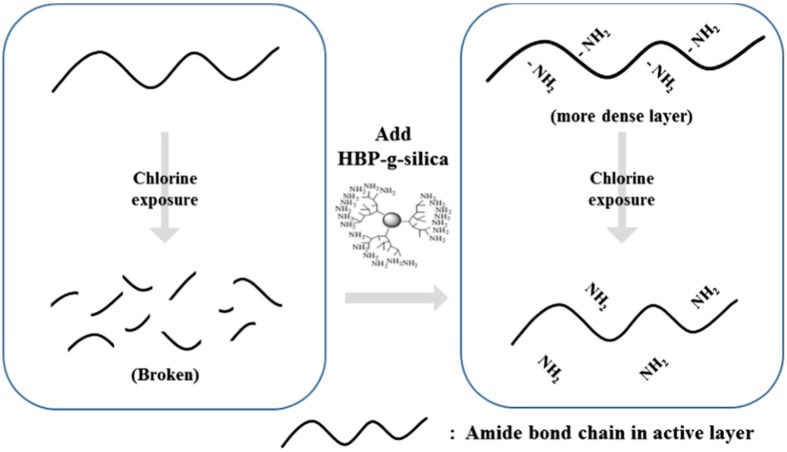
Protection sequence against chlorine attack, reproduced with permission from Kim et al. [85], with copyright permission from © 2013 Elsevier.

It is known that PA composite membranes are very sensitive to chlorine. As the PA layer touches the chlorinated water, the amine groups oxidize by chlorine and decompose in water, leading to deteriorated separation performance [108]. Park et al. [69] used acid functionalized MWCNTs to improve the chlorine resistance. When MWCNTs were incorporated into the PA active layer, the membrane showed enhanced anti-chlorine properties. This could be ascribed to the reaction between the functional groups in carbon nanotubes and the amine groups in PA structure, which as a result formed a barrier above the PA that reduced membrane chlorine exposure. Another study by Kim et al. [85] showed that attaching hyper branched polyamide modified silica NPs onto PA layer could protect the membrane from chlorine attack. The extra amino groups presented by the functionalized silica NPs were the main target for chlorine and subsequently lessened membrane surface exposure, as shown in Figure 4. It seems that all researchers followed the same strategy to produce a membrane with high chlorine resistance, which generates a protection layer on the membrane surface; however, this could not provide long term efficiency as the barrier might be finally degraded and the chlorine reaches the membrane surface [82,87,109].

Membrane fouling is generally known as the accumulation of unwanted materials on membrane surfaces [110]. Organic and micro-biological fouling of TFN membranes are among the substantial reasons that lead to membrane performance declination [111]. Hence, many studies are devoted to develop a TFC with desired fouling resistance without “trading off” any of the other properties including permeability and rejection efficiency. Kim et al. [112] showed that incorporating hydrophilic filler into the PA layer could increase membrane resistance against organic fouling. The results demonstrated that there was a reverse relationship between hydrophilicity and organic foulants accumulation. This could be ascribed to the weak interaction between organic foulants and hydrophilic surfaces. Another study by Rana et al. [75] exhibited that an increase in membrane’s negative surface charge could enhance fouling resistance, due to the strong repulsive force between the membrane and negatively charged foulants. Lee et al. [113] used Ag nanoparticles as fillers in the PA to mitigate bacterial accumulation on the surface. Results showed that the Ag-TFN membrane has better resistance against bacterial fouling. It is believed that Ag nanoparticles disturb the permeability and respiration functions of the bacterial cell, and eventually destroy the DNA [114,115,116].

**Figure 5:**
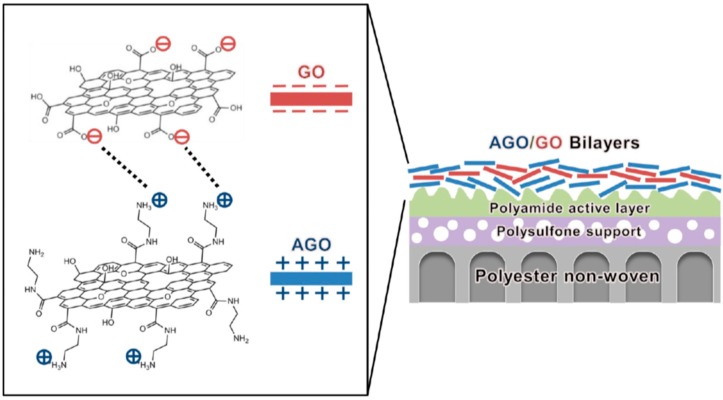
Layer-by-layer deposition of positively-charged GO and aminated-GO nanosheets on the membrane surface; reproduced with permission from Choi et al. [118], with copyright permission from © 2013, American Chemical Society.

**Figure 6:**
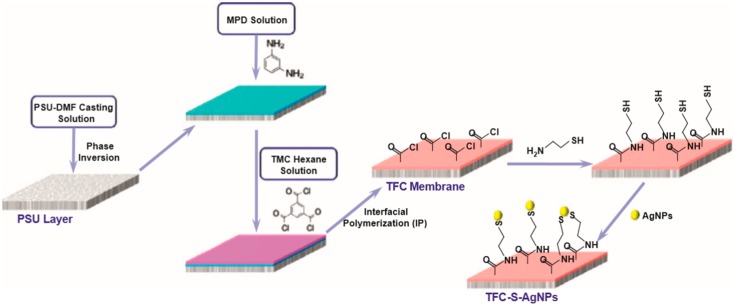
Schematic illustration of attaching Ag-NPs on the TFC membrane surface; reproduced with permission from Yin et al. [120], with copyright permission from © 2013 Elsevier.

Kim et al. used a new approach to prepare hybrid TFC by the self-assembly between titanium oxide NPs and PA’s carboxylic functional groups [117]. Results indicated that the UV irradiation of the membrane could reduce E-coli content on the surface and this was attributed to the ability of TiO_2_ to form different hydroxyl and peroxide radicals under the influence of UV light. These active radicals were capable of destroying the bacterial cells. Ben-Sasson et al. [116] used electrostatic attraction to attach copper (Cu) nanoparticles to the TFC membrane surface. Results indicated that the presence of positively charged Cu-NPs did not affect the overall hydrophilicity of the membrane, but reduced the growth of bacterial cells. The SEM images of the membrane’s surface exhibited that the bacterial cells were damaged when contacted with Cu-NPs. This could be ascribed to the high toxicity of Cu that led to bacterial DNA damage. Choi et al. [118] used “layer-by-layer assembly” to attach graphene oxide (GO) and aminated-graphene oxide (AGO) to TFC membrane surface, as shown in Figure 5. The resultant TFC showed enhanced resistance against organic fouling and chlorine attack, while preserving water flux and NaCl rejection. Hu and Mi [119] succeeded in using layer-by-layer deposition” to connect GO NPs to the PA. In this case, GO-NPs formed linkages with membrane’s functional groups. The newly developed membrane exhibited superior water flux and excellent dye rejection. The disadvantage of the “surface located nanocomposite membrane” is the loss of nanoparticles during filtration, especially those attached by electrostatic forces. The depletion of nanoparticles reduces the efficiency of the membrane and may expose nanoparticles to the permeated water causing a threat to people’s health. As a result, Yin and co-workers. [120] used cyseteamine as a “bridging agent” to attach silver (Ag) nanoparticles to the membrane surface, as shown in Figure 6. Results indicated that the modified membrane has stable Ag NPs, superior antimicrobial properties, high permeability, and good separation efficiency. Recently, Mayyahi [121] showed that UV irradiation of TFN membrane which impregnated with TiO_2_ could result in robust antibacterial properties.

## 6. Conclusions

A tremendous development in TFN membranes for water purification has been achieved including producing a membrane with super water flux, high salt rejection, and excellent fouling and chorine resistance via using innovative approaches such as imparting the favored properties of nanoparticles to the membrane surface, optimizing the membrane fabrication process, modifying the materials that are required to synthesis the membrane, and changing membrane surface properties by post-treatment. However, researchers have failed to find an alternative to the PA barrier layer or to suggest a new support layer. We do agree that PA atop PSU/PES showed robust efficiency in RO and other water treatments applications, but these membranes have been used since 1970 and scholars have successfully addressed almost all the challenges facing the progress of such membranes. Forthcoming researches should be dedicated to suggest a new reverse osmosis membrane rather than developing the existing one.

## Figures and Tables

**Table 1:** Reported monomers for synthesis of polyamide composite membranes.

Amine	Chemical Structure	Acid Chloride	Chemical Structure	Membrane Performance	Ref.
MPD	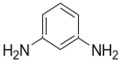	TMC	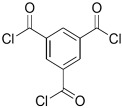	It is well-known that the interfacial polymerization of MPD and TFC on a porous support layer results in high water flux and salt rejection	[17]
BDSA	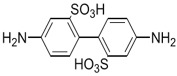	TMC	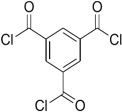	Water Flux increased by more than 100% by using BDSA in the interfacial polymerization. Simultaneously, salt rejection increased from 89 to 99%.	[24]
S-BAPS	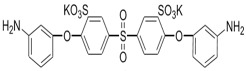	TMC	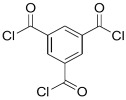	When compared to the traditional TFC membrane, this membrane showed higher water flux, but lower NaCl rejection and chlorine resistance.	[25]
BHDT	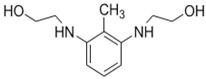	TMC	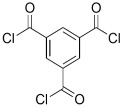	This membrane demonstrated higher chlorine resistance when compared to the normal TFC membrane.	[26]
PAMAM	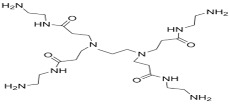	TMC	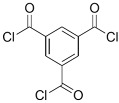	In this study, the effects of PAMAM content on TFC membrane performance were studied. NaCl rejection was increased when PAMAM concentration was increased from 0.1% to 0.5% (*w*/*v*), while water flux was reduced.	[27]
DETA, TETA, or TEPA	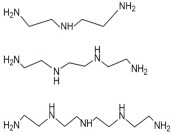	TMC	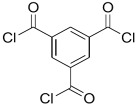	Under operating pressure of 36.52 psi, water fluxes of TEPA/TMC, TETA/TMC, and DETA/TMC were 51.1 ± 4.5, 43.5 ± 0.5, and 33.5 ± 2 L/m^2^·h, respectively. On the other hand, Na_2_SO_4_ rejection sequence was: DETA/TMC > TEPA/TMC > TETA/TMC.	[28]
DPA	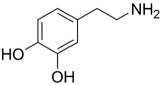	TMC	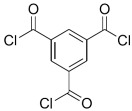	The polyester bonds of DPA/TMC produced TFC membrane with high chemical stability, while maintaining good performance.	[29]
DABA	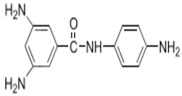	TMC	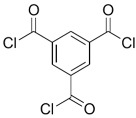	Results showed that as DABA concentration was increased, the membrane became more hydrophilic and as a result, high water flux (55.4 L/m^2^·h-250 psi) was achieved.	[23]
MPD	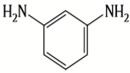	DMSO	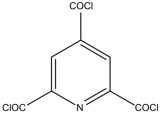	The developed membrane showed excellent antimicrobial efficiency and high water flux and salt rejection.	[30]
MPD	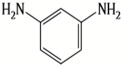	BTAC	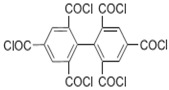	Membrane surface was highly negatively charged, smooth, and very thin, which in turn produced high fouling resistance.	[31]
SMPD	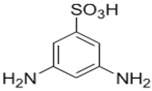	TMC	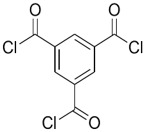	When SMPS content was increased, the molecular weight of PA was decreased, and it subsequently increased water flux and decreased NaCl rejection.	[32]
MPD	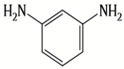	mm-PETC	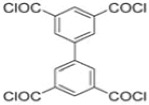	Under 290 psi, water flux was 37.1 L/m^2^·h and NaCl rejection was 98.4%	[22]
MPD	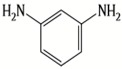	om-PETC	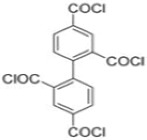	Under 290 psi, water flux was 50 L/m^2^·h and NaCl rejection was 97.8%	
MPD	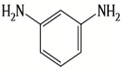	op-PETC	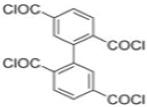	Under 290 psi, water flux was 45.2 L/m^2^·h and NaCl rejection was 97.2%	
MPD	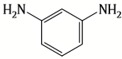	ICIC	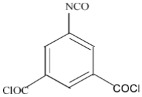	Under operating pressure of 232 psi, water flux was 63 L/m^2^·h and NaCl rejection was 98.2%. In addition, the membrane showed significant resistance against chlorine.	[33]
MPD	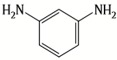	CFIC	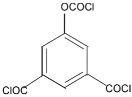	Under operating pressure of 232 psi, water flux was around 43.3 L/m^2^·h and NaCl rejection was around 98.6%. In addition, the membrane showed significant resistance against chlorine.	
HFA-MDA	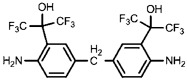	TMC	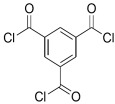	Under operating pressure of 400 psi, NaCl rejection was 85% at low pH 4, but increased to 96.1% at pH 10. Water flux was 48 L/m^2^·h and 80 L/m^2^·h at pH 4 and pH 10, respectively. Besides, the membrane showed significant chlorine resistance.	[34]
Bisphenol A	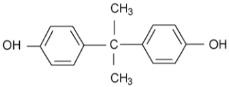	TMC	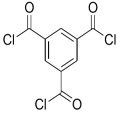	This membrane showed significant fouling resistance along with high water flux and salt rejection.	[35]
TMBPA	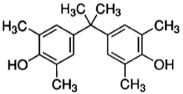	TMC	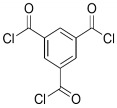	Under operating pressure of 130 psi, water flux was 66.7 L/m^2^·h and the membrane showed good antifouling properties.	[36]

**Table 2:** Summary of important fillers used to modify TFC membranes.

Nanofiller	PA Layer Monomers	Substrate	Performance of TFN	Ref.
Zeolite NaA	MPD-TMC	PSU	Water flux was increased from 2.5 × 10^12^ to 3.9 × 10^12^ mPa^−1^·s^−1^ without compromising salt rejection (94%) by increasing the concentration of nanoparticles from 0 to 0.4 wt.%.	[60]
Zeolite NaAAaA	MPD:TEA-TMC	PSU	Both AgA-TFN and NaA-TFN membranes exhibited higher water flux than that of TFC membrane. No change in salt rejection was observed. Both membranes showed enhanced antimicrobial properties.	[61]
Different sized zeolite	MPD:TEA:SLS:IPA-TMC	PSU	The membrane embedded with smaller zeolite NPs produced higher water flux than the membrane with larger zeolite NPs.	[62]
Silica	MPD-TMC	PSU	By increasing silica concentration, the thermal properties of the membrane were considerably enhanced.	[68]
MWCNTs	MPD-TMC	PSU	Under filtration pressure of 225 psi, both water flux and salt rejection were decreased from 18 to 12 L/m^2^·h and 98 to 92.2 wt.%, respectively, by increasing the concentration of MWCNTs from 0 to 1 wt.%. On the other hand, the membrane demonstrated significant chlorine resistance.	[69]
Zeolite -LTA	MPD-TMC-post Treatment	PSU	NaCl rejection and water flux were 99.4 wt.% and 42 L/m·h, respectively, and had a filtration pressure of 300 psi.	[70]
F-Silica	MPD-TMC	PSU	When NPs concentration was 0.4 wt.%, the membrane showed high thermal stability.	[71]
F-MWCNTs	MPD-TMC	PSU	The membrane showed high dyes and brilliant blue rejection (91%)	[72]
Metal alkokxide	MPD: SLS-TMC	PSU	Water flux was encreased by approximately 2-fold when compared with the virgin membrane.	[73]
Zeolite NaX	MPD-TMC	PES	Under filtration pressure of 175 psi, the water flux was increased from 8.01 to 29.76 L/m^2^·h by increasing the content of NPs from 0 to 0.2 wt.% without jeopordizing NaCl rejection (above 90%). Also, the membrane showed good thermal stability.	[74]
iLSMM	MPD-TMC	PSU	Under filtration presure of 300 psi, the optimized water flux was 42 L/m^2^·h and the NaCl rejection was 97%. Besides, the membrane showed good antifouling properties.	[75]
MCM-41	MPD-TMC	PSU	Under filtration pressure of 300 psi, Water flux was increased from 28 to 46 L/m^2^·h by increasing the concentration of NPs from 0 to 0.1 wt.%, while NaCl rejection was maintained (97 wt.%).	[76]
APQZ	MPD-TMC	PSU	Water flux was increased from 16 to 40 L/m^2^·h by increasing the concentration of NP from 0 to 0.1 wt.%. In addition, the membrane showed good mechanical stability.	[77]
Zwitterion-CNT	MPD-TMC	PES	Under 530 psi, the optimized water flux was 48.46 L/m^2^·h, and NaCl rejection was 98.6%.	[78]
Carboxylic MWNTs	MPD-TMC	PES	Under 100 psi, the optimized water flux was 40 L/m^2^·h. Moreover, the membrane showed good mechanical stability.	[79]
Zeolite (Silicate-1)	MPD-TMC	PSU	The membrane showed higher chemical stability than the one with NaX-Zeolite NPs.	[80]
Zeolite-NaA	MPD-TMC	PSU	Under 232 psi, good water flux was achieved (46.5 L/m^2^·h) by adding the NPs in organic phase and high salt rejection (97%) by adding the NPs in aqueous phase.	[81]
Aminated Zeolite	MPD:aPES:TEA-TMC	PSU	Under 797 psi, adding PES and TEA to MPD-nanoparticle solution increased water flux from 23.2 to 37.8 L/m^2^·h without compromising salt rejection (98%). Moreover, the membrane showed good chlorine resistance.	[82]
Zeolite-A	MPD-TMC	PSU	The membrnae showed significant fouling resistance.	[83]
Mesoporous SiO_2_	MPD-TMC	PSU	Under 232 psi, water flux was increased from 19 to 53 L/m^2^·h by increasing the concentration of NPs from 0 to 0.1 wt.%, while NaCl rejection remained (97%).	[84]
HBP-g-silica	MPD: aPES-TMC	PSU	Under 797.7 psi, the optimized water flux was 34.4 L/m^2^·h, while the salt rejection was 97.7%. And, the membrane showed better chlorine resistance.	[85]
Aluminosilicate CNTs	MPD-TMC	PSU	Under 232 psi, the optimized water flux was 23 L/m^2^·h, while NaCl rejection was 97.5%.	[86]
F-MWCNTs	MPD-TMC	PSU	Under 232 psi, the optimized water flux was 28.05 L/m^2^·h, while salt rejection was 90%. In addition, the membrane showed better antifouling and antioxidant properties.	[87]
HNTs	MPD-TMC	PSU	Under 217.5 psi, water flux was increased from 18 to 36.1 L/m^2^·h by increasing the concentration of NPs from 0 to 0.1% without sacrificing NaCl rejection (93%). Besides, the membrane had enhanced fouling properties.	[88]
OA-SiO_2_	MPD-TMC	PSU	The OA modified-silica PA membrane produced higher salt rejection (98%) when compared to the unmodified silica PA membrane (95%).	[89]
Clay	MPD-TMC	PSU	Under 232 psi, water flux was increased from 36.6 to 51 L/m^2^·h by adding 0.1 wt.% NPs without compromising NaCl rejection (around 99%). Also, the membrane exhibited significant antifouling properties.	[90]
GO-TiO_2_	MPD-TMC	PSU	Under 217.5 psi, both water flux and salt rejection were increased from 34 to 51 L/m^2^·h and 97 to 99%, respectively, by adding 0.02 wt.% NPs. Besides, the membrane demonstrated robust chlorine resistance.	[91]
HN_2_-TNTs	MPD-TMC	PSU	Under 217.5 psi, both water flux and NaCl rejection were increased from 19 to 36 L/m^2^·h and 94 to 96%, respectively, by adding 0.05 wt.% NPs. Moreover, the membrane showed good fouling resistance.	[92]
GO	MPD-TMC	PSU	Under 217 psi, the optimized water flux was 22 L/m^2^·h, while NaCl rejection was above 80%. Moreover, the modified membrane exhibited excellent fouling resistance against BSA and HA.	[93]
Al-ZnO	MPD-TMC	PSU	Under 225 psi, the optimized water flux was 32 L/m^2^·h, while NaCl rejection was 98%.	[94]
MCM-48-SiO_2_	MPD-TMC	PSU	Under 232 psi, the optimized water flux was 68 L/m^2^·h. And, NaCl rejection was around 97%.	[95]
GO	MPD-TMC	PSU	Under 300 psi, water flux was increased from 39 to 60 L/m^2^·h by increasing NPs concentrations from 0 to 0.015 wt.%, while NaCl rejection was above 93%.	[96]
ZnO	MPD-TMC	PSU	Under 300 psi, water flux was increased from 60 to 85 L/m^2^·h by increasing the concentration of ZnO from 0 to 0.1 wt.%. Under UV irradiation the membrane showed super water flux (120 L/m^2^·h). In addition, the membrane showed excellent fouling resistance.	[97]
MOFs	MPD-TMC	PSU	Under operation pressure of 300 psi, water flux and NaCl rejection were 85 L/m^2^·h and 98.5%, respectively.	[98]
Graphene quantum dots	PIP-TMC	PES	Under operation pressure of 0.2 Mpa, water flux was 120 L/m^2^·h, 6.8-times higher than that of the virgin membrane. Moreover, the membrane showed excellent fouling resistance.	[99]
ZIF-8	MPD-TMC	PSU	53% enhancement in water flux was achieved. NaCl rejection was 99.4%.	[100]
TiO_2_	MPD-TMC	PES	The addition of TiO_2_ resulted in higher water flux (24.3 L/m^2^·h) as compasred with the virgin TFC (21.5 L/m^2^·h), while membrane selectivity was preserved (97%). Additionally, by increasing feed solution temeprature from 25 to 65 °C, further enhancement in water flux was achieved.	[101]
CQDs	PIP-TMC	PSU	The addition of carbon quantum dots led to significant incerease in permeate flux (from 18 to 42.1 L/m^2^·h) without jeopordizing Na_2_SO_4_ rejection (93%). Moreover, the fouling capacity of membrane was enhanced.	[102]
Na^+^ functionalized CQDs	MPD-TMC	PES	Impresive water flux (104 L/m^2^·h), high rejection of SeO_3_^2^ (97.5%), and excellent fouling resistance were achieved when quantum dots concentration was 0.05 wt.%.	[103]
SiO_2_	MPD-TMC	PSU	Water flux was increased from 30 to 50 L/m^2^·h by increassing NPs concentration from 0 to 0.1 wt.% along with slight increase in salt rejection (from 92 to 95%).	[104]
Ziconiumv (IV)-carboxylate MOFs	MPD-TMC	PSU-PVP-LiCl	52% increase in water flux was achieved without comprimising NaCl rejection (95.5%).	[105]

## Abbreviations

AGO	Aminated-graphene oxide
Ag	Silver
Al-ZnO	Aluminum doped zinc oxide
BDSA	2,2′-benzidinedisulfonic acid
BHAC	2,2′,4,4′,6,6′-biphenyl hexaacyl chloride
BHDT	Bis-2,6-*N*,*N*-(2-hydroxyethyl) diaminotoluene
BPA	Bisphenol
CFIC	5-chloroformyloxy-isophthaloyl chloride
CNT	Carbon nanotube
CQDs	Carbon quantum dots
Cu	Copper
DABA	Triamine 3,5-diamino-*N*-(4-aminophenyl)-benzamide
DMF	*N*,*N*-dimethylformamide
DMSO	2,4,6-pyridinetricarboxylic acid chloride
DETA	Diethylenetriamine
DPA	Dopamine
F-MWCNTs	Functionalized multi wall carbon nanotubes
F-silica	Functionalized silica
GO	Graphene oxide
HBP-g-silica	Hyper-branched aromatic polyamide-grafted silica
HNTs	Halloysite nanotubes
ICIC	5-isocyanato-isophtahloyl chloride
IP	Interfacial polymerization
iLSMM	In-situ hydrophilic surface modifying macromolecules
MOFs	Metal–organic frameworks
MPD	m-phenylenediamine
mm-BTEC	3,3′,5,5′-biphenyl tetraacyl chloride
MWCNTs	Multiwall carbon nanotubes
NPs	Nanoparticles
OA-SiO_2_	Oleic acid modified silica
om-BTEC	2,2′,4,4′-biphenyl tetraacyl chloride
MOFs	Metal–organic framework
op-BTEC	2,2′,5,5′-biphenyl tetraacyl chloride
PA	Polyamide
PAMAM	Ethylenediamine cored poly(amidoamine)
PDMAEMA	Poly(*N*,*N*-dimethylaminoethyl methacrylate)
PSU	Polysulfone
RO	Reverse osmosis
SiO_2_	Silicon dioxide
SMPD	Sulfonated m-phenylenediamine
SWCNTs	Single-wall carbon nanotubes
TFC	Thin film composite
TFN	Thin film nanocomposite
TiO_2_	Titanium dioxide
TMC	Trimesoyl chloride
TETA	Triethylenetetramine
TEPA	Tetraethylenepentamine
TNTs	Titanate nanotubes
UV	Ultraviolet
ZIF	Zeolitic imidazolate framework
ZnO	Zinc oxide
